# Overexpression of *OCT-1* gene is a biomarker of adverse prognosis for diffuse large B-cell lymphoma (DLBCL): data from a retrospective cohort of 77 Brazilian patients

**DOI:** 10.1186/s12885-020-07553-2

**Published:** 2020-10-29

**Authors:** Gisele R. Gouveia, Suzete C. Ferreira, Sheila A. C. Siqueira, Luis Alberto de Pádua Covas Lage, Abrahão E. Hallack Neto, Renata de Oliveira Costa, Juliana Pereira

**Affiliations:** 1 Laboratory of Medical Investigation in Pathogenesis and Directed Therapy in Onco-Immuno-Hematology (LIM-31), Faculty of Medicine, University of Sao Paulo’s Medical School (FM-USP), Av. Dr. Enéas Carvalho de Aguiar, 155, Cerqueira César, São Paulo Brazil; 2 Department of Molecular Biology, Pró-Sangue Foundation, Sao Paulo Blood Bank, São Paulo, Brazil; 3 Department of Pathology, Hospital das Clínicas – Faculty of Medicine, Sao Paulo University (HC-FM-USP), São Paulo, Brazil; 4 Department of Hematology, Hemotherapy and Cell Therapy, Faculty of Medicine, Sao Paulo University (FM-USP), São Paulo, Brazil; 5 Department of Hematology and Hemotherapy, University of Juiz de Fora (UJF), Juiz de Fora, Brazil; 6 Department of Hematology and Hemotherapy, Centro Universitário Lusíadas (FCMS/UNILUS), Santos, Brazil; 7 Laboratory of Medical Investigation in Pathogenesis and Directed Therapy in Onco-Immuno-Hematology, Nucleus of non-Hodgkin’s Lymphomas & Histiocytic Disorders (LIM-31/FM-USP), São Paulo, Brazil

**Keywords:** Diffuse large B-cell lymphoma (DLBCL), *OCT-1* gene, *BCL-2* gene, Prognosis, Immunochemotherapy

## Abstract

**Background:**

*OCT-1* gene is a member of the POU-homeodomain family of transcriptional regulators of B-lymphocyte differentiation by controlling expression of B-cell specific genes. *BCL-2* gene is a potent inhibitor of apoptosis and it is essential during B-cell differentiation into germinal center. These genes may be expressed in diffuse large B-cell lymphoma (DLBCL), but the role of *BCL-2* in its prognosis has been contradictory, and *OCT-1* has yet to be tested.

**Methods:**

In this study, we aimed to investigate the prognostic impact of *OCT-1* and *BCL-2* expression in DLBCL treated in the real world with immunochemotherapy in a single center. *BCL-2* and *OCT-1* genes were available in 78.5% (77/98) DLBCL patients, and the RNA for quantitative real-time PCR was isolated from formalin-fixed paraffin-embedded samples. The values obtained for gene expression were transformed in categorical variable according to their median.

**Results:**

Cohort median age was 54.5 years (15–84), 49 (50%) were male, 38/77 (49.4%) and 40/77 (51.9%) presented *OCT-1* and *BCL-2* expression ≥ median, respectively. The overall response rate (ORR) in all patients was 68.4% (67/98), 65,3% (64/98) of patients acquired complete response, and 3.1% (3/98) partial response, while 6.1% (6/98) were primary refractory. The median follow-up was 3.77 years (95% CI: 3.2–4.1), with 5.43 (95% CI: 2.2-NR) of overall survival (OS) and 5.15 years (95% CI: 2.9-NA) of progression free survival (PFS). *OCT-1* ≥ median was associated with shorter OS at univariate analysis (*p* = 0.013; [HR] 2.450, 95% CI: 1.21–4.96) and PFS (*p* = 0.019; [HR] 2.270, 95%CI: 1.14–4.51) and *BCL-2* gene overexpression presented worse PFS (*p* = 0.043, [HR] 2.008, 95% CI: 1.02–3.95). At multivariate analysis, *OCT-1* overexpression was associated with poor PFS (*p* = 0.035, [HR] 2.22, 95% CI: 1.06–4.67).

**Conclusion:**

In this study, we showed that overexpression of *OCT1* gene was an independent prognostic factor of adverse outcomes in DLBCL.

## Background

Diffuse large B-cell lymphoma (DLBCL) is the main subtype of non-Hodgkin’s lymphoma (NHLs) [1] and represents 49.5% of all NHLs in our institution [2]. Immunochemotherapy with rituximab 375 mg/sqm, cyclophosphamide 750 mg/sqm, doxorubicin 50 mg/sqm, and vincristine 1.4 mg/sqm (maximun at 2 mg) intravenously on day 1, plus oral prednisone 100 mg/sqm on days 1–5 (R-CHOP) was established as the gold standard of treatment for DLBCL [3]. However, more intensive regimens combining additional antineoplastic agents given in a continuous infusion such as DA-REPOCH have improved the overall survival of mediastinal primary lymphoma (PMBCL) and high grade lymphoma with *BCL-2* and *MYC* genes rearrangement with or without *BCL-6* rearrangement [4, 5].

The addition of the monoclonal antibody anti-CD20 to the CHOP regimen provided significant improvement in the survival of DLBCL patients [3, 6, 7]. However, despite this considerable advance, half of DLBCL patients treated with R-CHOP remain incurable [8] with a median 5-year overall survival (OS) and progression-free survival (PFS) of 53 and 89%, respectively [8]. Unfortunately, after introduction of rituximab to the CHOP backbone, clinical prognostic index as the International Prognostic Index (IPI) has failed to stratify different prognosis in the group with OS inferior to 50% [9]. The recent NCCN International Prognostic Index (NCCN-IPI) established by Zhou et al., showed better capacity to discriminate a high-risk population than IPI [10, 11]. However, the determination of the biological heterogeneity of DLBCL to improve risk prediction and design targeted therapies for poor prognosis is an urgent need.

*BCL-2* is an antiapoptotic gene located on chromosome 18 generally expressed in many normal cells, as well as in different neoplasms, especially in follicular lymphoma and DLBCL [12]. Its prognostic value in DLBCL patients has been contradictory since the introduction of rituximab to the CHOP regimen [13]. The Octamer transcription factor 1 (*OCT-1*)/*POU2F1* gene is a master regulator of B-cell differentiation [14] and its transcription encodes the histone protein H2B. It belongs to the POU-homeodomain transcription factor family with a highly conserved DNA-binding domain that modulates specific genes of B-cell lineage, cell survival and cell proliferation [14, 15]. *OCT-1* has been described as a contributor for malignant transformation in different tumors, such as in gastric cancer [16], prostate [17] and cervical cancer [18]. The inhibition of *OCT-1* and *OCT-2* expression by RNA interference promotes apoptosis and down-regulation of *BCL-2* gene expression [19]. However, few studies have investigated *OCT-1* and *BCL-2* gene in malignant lymphoma in the same cohort [20]. A high content of Oct-1 protein in tumors confers poor prognosis. Overexpression of Oct-1 in gastric tumors conferred worse prognostic with 5-year OS of 8.9% in comparison to 51.1% in cases with normal levels of Oct-1 expression in tumor [18].

Previously, our group described that high expression of Bcl-2 and p63 proteins determined worse prognosis in DLBCL treated with anthracycline-based chemotherapy without rituximab [21, 22]. In this study, we aimed to assess the role of *BCL-2* and *OCT-1* gene expression by quantitative real time PCR (qRT-PCR) in the prognosis of DLBCL patients treated with immunochemotherapy in a single center in Brazil.

## Methods

### Patients

In this retrospective study, tumor samples and clinical data of patients were obtained from a single general hospital in Latin America. Patients were identified in different data banks and the clinical features were collected directly from medical records and tumor samples used were kept in the archives of the Pathology Department. After the first screening, 98 patients with de novo DLBCL treated between January 2006 and January 2011 were identified. The molecular and immunohistochemistry analysis were performed on 77 patients in this cohort (77–98), which had sufficient material to carry out these tests. The study was approved by the Ethics Committee for Research Project Analysis at our institution, and performed in agreement with the Declaration of Helsinki. All cases were centrally reviewed by a hematopathologist and classified according to the WHO 2008.

According to the protocol settled in the period of the study, patients were staged by Ann Arbor system using clinical examination and computerized tomography (CT) scan of the neck, thorax, abdomen and pelvis. In patients with central nervous system symptoms, brain magnetic resonance imaging and lumbar puncture were indicated. Digestive endoscopy was also indicted in case of gastric symptoms, and the Waldeyer’s ring involvement by lymphoma. Patients should have received a minimum of six, and a maximum of eight cycles of R-CHOP 21 (rituximab 375 mg/sqm intravenously (iv) on day 1 (D1), cyclophosphamide 750 mg/sqm iv on D1, doxorubicin 50 mg/sqm iv on D1, vincristine 1.4 mg/sqm iv [maximum 2 mg] on D1, and prednisone 100 mg p.o. on D1–D5). Involved-field radiotherapy with 36 Gy was given on *bulky* region for patients with stage I/II and with extranodal lymphoma. Central nervous system prophylaxis was done with four doses of intrathecal methotrexate 12 mg and dexamethasone 2 mg at D1 of cycles 1, 2, 3 and 4 in patients with lymphoma of the testis, breast, sinuses and presence of paravertebral mass.

The interim response and the end of treatment response analysis followed the International Workshop to Standardize Response Criteria for Non-Hodgkin’s Lymphoma (IWRC) criterion [23, 24]. Patients were reassessed after four cycles and after the last cycle of chemotherapy or radiotherapy with neck, thorax, abdomen and pelvis CT scans. Afterwards, CT scans were performed every 16 and 24 weeks in the first and second years of follow up respectively, and subsequently with clinical examination every 24 weeks and so forth.

The end points used to test the biological and clinical variables were overall survival (OS) and progression free survival (PFS). The regularly tested clinical variables in this study were age, gender, lactate dehydrogenase (LDH), performance status, B symptoms, and bulky disease characterized as tumor size ≥10 cm, bone marrow infiltration, disease stage and IPI score.

### Histological diagnosis and immunohistochemistry

In all cases (*N* = 98) the diagnosis was established based on the histopathological analysis of lymph nodes or extra-lymphoid tissues involved by the neoplasm. Microscopic analysis was performed in optical microscope (Olympus BX51TF, magnification of 200x and 400x – Olympus Plan N) and images were captured by Olympus C-7070 CAMEDIA photographic camera). Hematoxylin-Eosin (H&E) staining demonstrated an atypical lymphoid infiltrate composed of large cells that infiltrated the lymph nodes in a diffuse manner with complete rupture of the nodal architecture, in addition to the presence of mitosis figures and occasional tumor necrosis. The diagnostic confirmation was based on immunohistochemical staining, where the following markers were used: Ki67 (Dako, clone K55, 1: 1600), CD20 (Dako, clone L26, 1: 1000), CD3 (Dako, clone F7.2.38, 1: 500), CD10 (Novocastra, clone S6C6, 1: 2000), BCL-6 (Abcam, clone SP155, 1: 1500) and MUM-1 (Abcam, clone EP5699, 1: 500). All cases presented high index Ki 67 (≥40%), diffuse positivity for CD20 and negativity for CD3. The CD10, BCL-6 and MUM-1 markers were used to classify these lymphomas into type germinal center (GC) and type B activated cells (ABC), according to the algorithm of Hans et cols. Molecular analysis was not used in this study to classify these lymphomas.

The immunohistochemical (IHC) staining for Bcl-2 and Oct-1 proteins was performed by immunoperoxidase technique using anti-Bcl-2 monoclonal antibody at dilution of 1/400 (Zeta, clone E-17) and anti-Oct-1 polyclonal antibody at dilution of 1/100 (Abcam), and diaminobenzidine immunoperoxidase (Dako, Denmark) was used to visualize the reaction. The slides were examined in an optic microscope by counting 500 cells, where Bcl-2 expression was considered positive when ≥30% of tumor cells stained positive for Bcl-2. Oct-1 positivity was graduated in a scale of 1+, 2+ and 3+ [25].

### Molecular biology

An unmodified form of mRNA was obtained from 5 μm sections of formalin-fixed paraffin-embedded (FFPE) tissue specimens obtained at diagnosis using the commercial kit RecoverAll Total Nucleic Acid Isolation (Ambion Inc., Austin, TX, USA) optimized in our laboratory as previously described [26]. Reverse transcription reaction was carried out with the commercial kit SuperScript III (Invitrogen Corporation, Carlsbad, CA, USA) according to the manufacturer’s instructions. Gene expression tests were accomplished by relative quantitative Real-Time PCR (qRT-PCR) with TaqMan Universal PCR Master Mix system (Applied Biosystems, Foster City, CA, USA) in StepOne Plus™ equipment (Applied Biosystems, Foster City, CA, USA). The *PRKG1* and *GAPDH* genes were standardized as reference for normalization of the genes of interest after a set of experiments [27, 28]. A cDNA pool containing five samples of reactive tonsil obtained from five different individuals was used to calibrate the samples. After data normalization [27, 28], the median expression of *BCL-2* and *OCT-1* genes were calculated as previously established [29].

The oligonucleotide sequences for the *BCL-2* gene were F: TTGCTTTACGTGGCCTGTTTC; R: GAAGACCCTGAAGGACAGCCAT. For the *OCT-1* gene, forward primer was 5′-CAGTGCAGCAACTACCCTCA-3′, and reverse primer was 5′-GGAGTGGAGGTGGTCTGTGT-3′. The authenticity and specificity of the primers was verified as RNA for humans in Primer-BLAST tool of the National Center for Biotechnology Information (NCBI), and then synthesized by Life Technologies (Invitrogen Corporation, Carlsbad, CA, USA). The probe FAM-ACCAGGAGGGGAAGGCCCCACAG-TAMRA was designed in different exons reducing the chance of contamination with genomic DNA using the Primer Express Software from Applied Biosystems, Foster City, CA, USA and then synthesized by Invitrogen Corporation (Carlsbad, CA, USA).

### Statistical analysis

The clinical and laboratorial features and gene expression results obtained from the study cohort were displayed in absolute and relative frequencies. The association among them was verified by the Fisher or likelihood ratio or X^2^ tests [30]. The normality distribution of the population tested for gene expression was verified by Shapiro–Wilk. Overall survival and progression free survival were estimated by Kaplan-Meier, and hazard ratios (HR) by Cox regression bivariate with 95% of confidence interval (95% CI) [31]. The statistically significant variables at univariate analysis were subsequently analyzed by multiple Cox regression [31]. The analysis was performed in the SPSS Statistics 20.0 software considering a *p*-value ≤5% as significant.

## Results

Among the 98 patients initially selected and confirmed as being DLBCL, 77 (78.5%) patients could be examined for *BCL-2* and *OCT-1* gene expression by qRT-PCR and 58 (59.2%) were available for IHC. The main cause for the missing data was lack of available tumor tissue and RNA degradation and consequent absence of amplification.

**Fig. 1:**
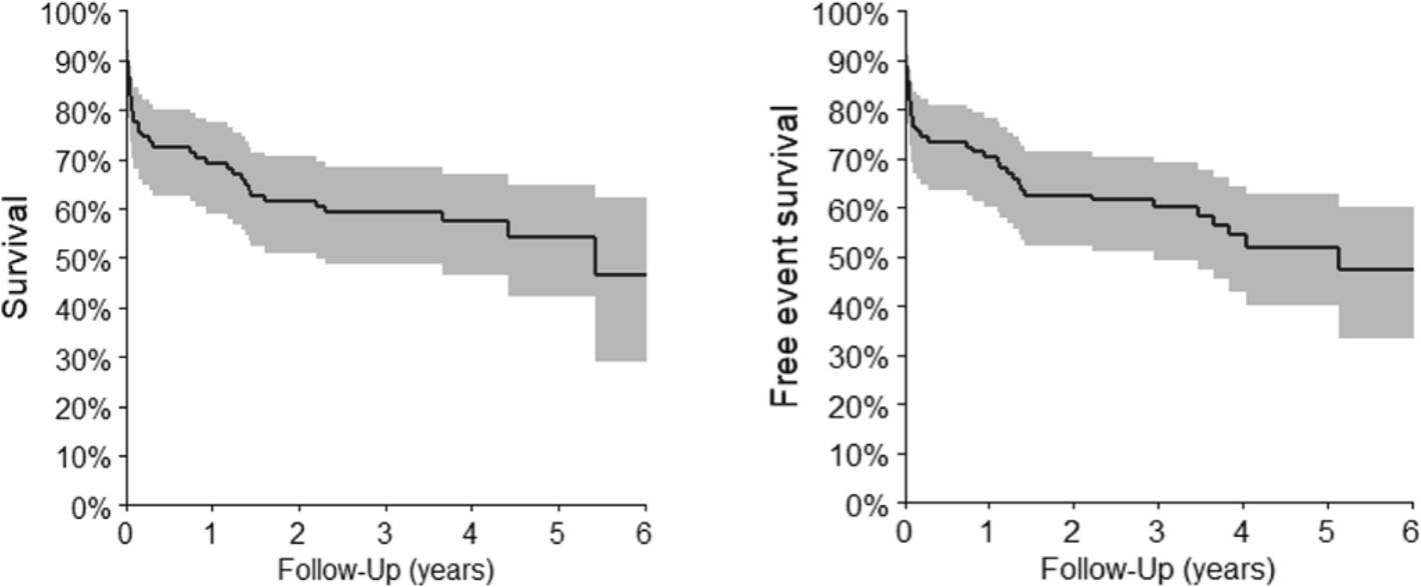
Overall survival (OS) and progression free survival (PFS) of the 98 Brazilian DLBCL patients

For all the cohort of 98 patients, the median age was 54.5 years (range 15 to 84 years), being 50% (49/98) male (Table 1). The overall response rate (ORR) was 68.4% (67/98), 65,3% of (64/98) patients acquired complete response (CR) and 3.1% (3/98) partial response (PR), 6.1% (6/98) of patients were primary refractory. The follow-up median was 3.77 years (95% CI: 3.2–4.1) and 25% (25/98) of patients died during the first line therapy period. The median OS and PFS was 5.43 years (95% CI: 2.2-NR) and 5.15 years (95% CI: 2.9-NR), respectively. The 5-year OS and PFS was 54.2% (42.2–64.8%) and 52.0% (40.1–62.6%), respectively (Fig. 1).

**Table 1:** Clinical features in 98 DLBCL Brazilian patients

	***n = 98***
Male, n (%)	49 (50.0)
Age, median (IQR)	54.5 (46.2–66.7)
BM+, n (%)	13 (13.3)
CNS+, n (%)	2 (2.0)
Extranodal sites ≥2, n (%) ^a^	7 (7.1)
ECOG > 2, n (%) ^b^	50 (50.0)
Bulky disease, n (%) ^b^	44 (44.9)
B symptoms, n (%) ^a^	66 (67.3)
Stage, n (%) ^c^	
I	10 (10.2)
II	30 (30.6)
III	13 (13.2)
IV	41 (41.8)
IPI, n (%) ^d^	
Low risk	24 (24.5)
Low-intermediate	20 (20.4)
Intermediate-high	22 (22.4)
High risk	27 (27.5)

Missing data:

^a^ *n* = 6 (6,1%)

^b^ *n* = 8 (8,1%)

^c^ *n* = 4 (4,1%)

^d^ *n* = 5 (5,1%)

The effect of *OCT-1* and *BCL-2* genes was measured as a categorical variable (cut-off point at median of expression). Then, patients were categorized in groups with gene expression below the median, and equal to or greater than the median. The values of OCT-1 expression were: mea*n* = 45.04 ng, median = 24.49 ng, range = 0.0–222.24 ng and for BCL-2 gene, the obtained values were: mean = 16.74 ng, media*n* = 6.27 ng, range = 0.0–102.79 ng.

No relevant differences were seen for clinical variables such as IPI score, gender, age, ECOG and disease stage between the *BCL-2* gene ≥ median or < median groups. In univariate analysis, *BCL-2* overexpression verified in 51.9% (*n* = 40/77) of cases was not statistically significantly associated with OS (*p* = 0.068). However, *BCL-2* expression ≥ median was associated with shorter PFS; the median PFS was 1890 days (63 months) for *BCL-2* < median and 1350 days (45 months) for *BCL-2* ≥ median. At 5-year, PFS was 64% for *BCL-2* < median and 38% for *BCL-2* ≥ median (*p* = 0.043; [HR] 2.008; 95% CI: 1.02–3.95).

**Fig. 2:**
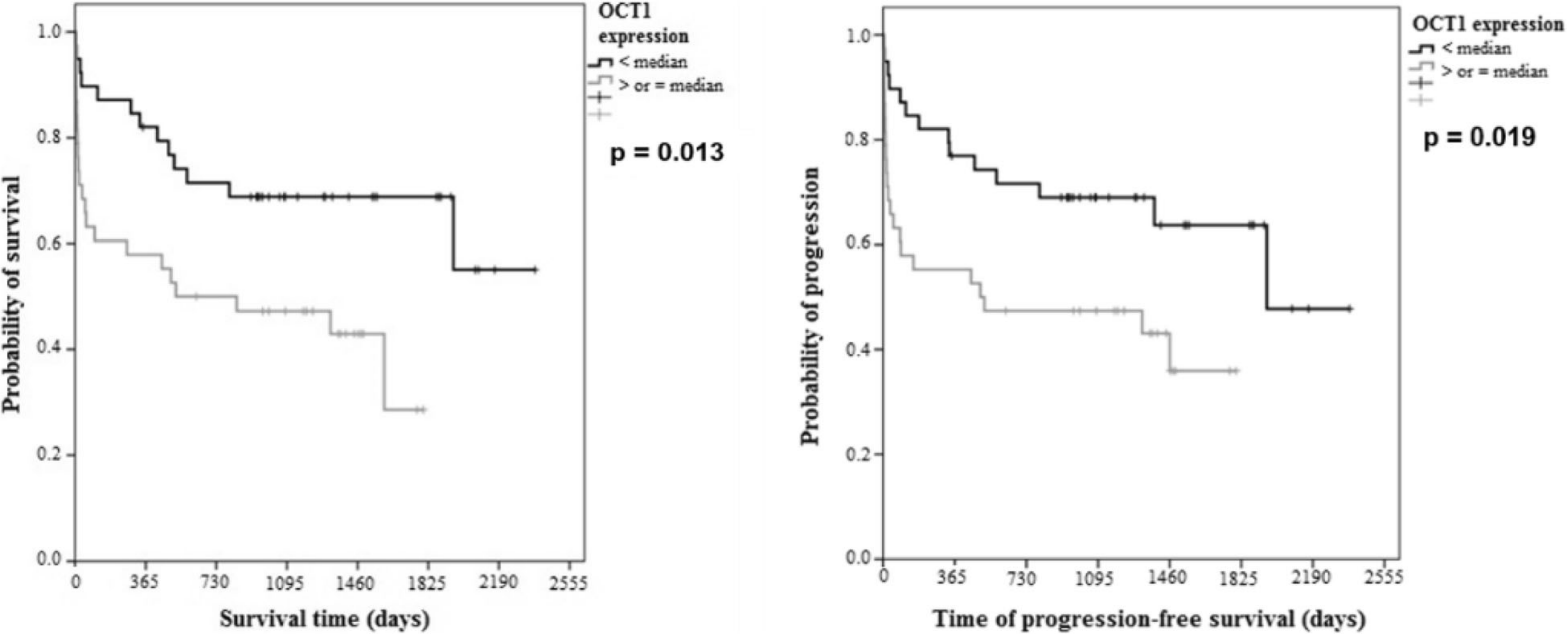
Overall survival (OS) and progression free survival (PFS) according to *OCT-1* gene expression

**Fig. 3:**
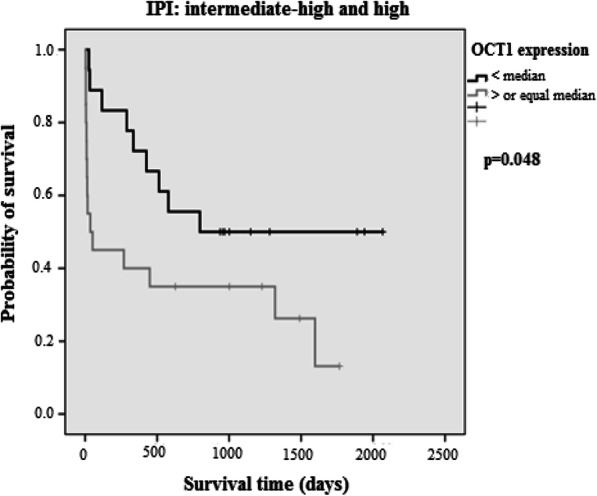
Overall survival (OS) in the intermediate-high and high-risk IPI subgroup according to *OCT-1* gene expression

**Fig. 4:**
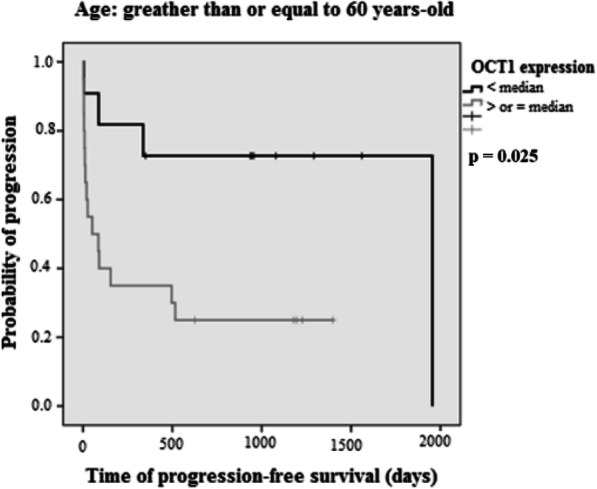
Progression-free survival (PFS) in the DLBCL patients ≥60 years subgroup according to *OCT-1* gene expression

*OCT-1* overexpression was verified in 49.4% (*n* = 38/77) of cases, predominantly in the subgroup of age ≥ 60 years (*p* = 0.029). The 5-year OS was 69% for *OCT-1* < median and 30% for OCT-1 ≥ the median; the median estimated OS was not reached for *OCT-1* < median and was 840 days (28 months) for *OCT-1* ≥ median (*p* = 0.013; [HR] 2.45, 95% CI: 1.21–4.96). The median of 5-year PFS was 63% for *OCT-1* < median and 37% for *OCT-1* ≥ the median; the median of PFS was 1950 days (65 months) for *OCT-1* < median and 510 days (17 months) for *OCT-1* ≥ median (*p* = 0.019; [HR] 2.27; 95% CI: 1.14–4.51) (Fig. 2). In the subgroup analysis, *OCT-1* overexpression was associated with worse OS (*p* = 0.048) in the high-risk aIPI subgroup (Fig. 3), and inferior PFS (*p* = 0.025) in patients ≥60 years (Fig. 4).

We analyzed the influence of OCT-1 gene expression level (< or ≥ median) in relation to the patient’s response regarding the treatment employed. Using the chi-square test, we found that the overall response rate (complete response + partial response) was higher in the OCT-1 group < median (33 of 39), when compared to the OCT-1 group ≥ median (20 of 38), in a statistically significant way (*p* = 0.005), thus validating the hyperexpression of the OCT-1 gene as an adverse prognostic factor in DLBCL.

### Immunohistochemistry

**Fig. 5:**
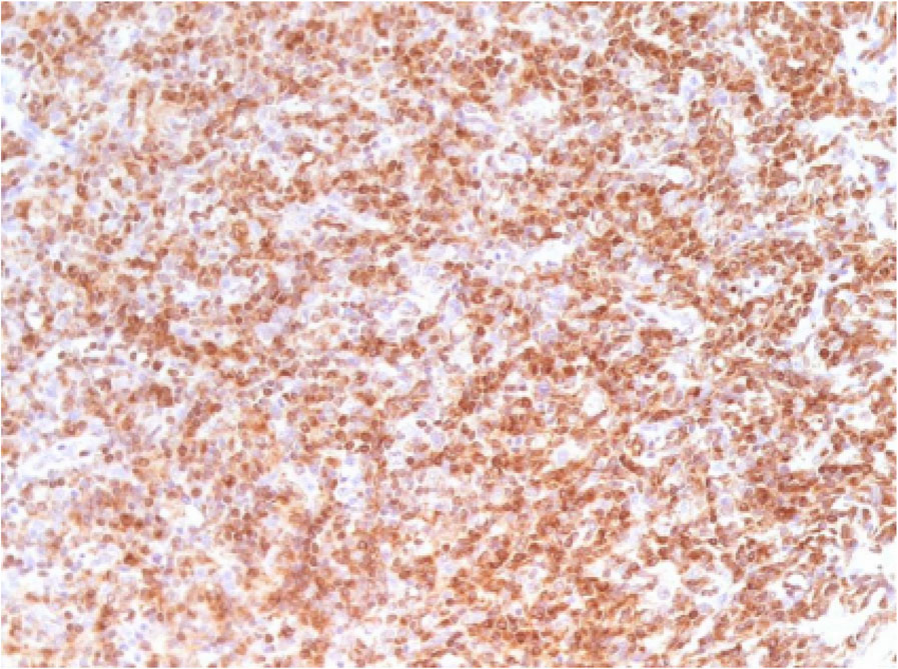
Immunohistochemical reaction showing positive expression of the BCL-2 protein. Optical microscopy, 200x magnification

**Fig. 6:**
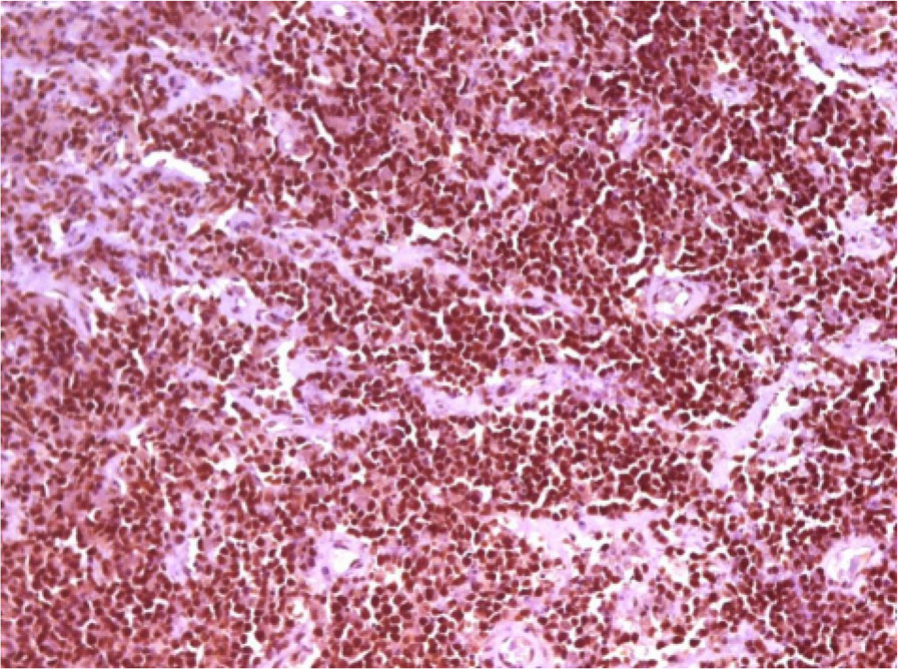
Immunohistochemical reaction showing moderate positive expression (2+/3+) of the OCT-1 protein. Optical microscopy, 200x magnification

**Fig. 7:**
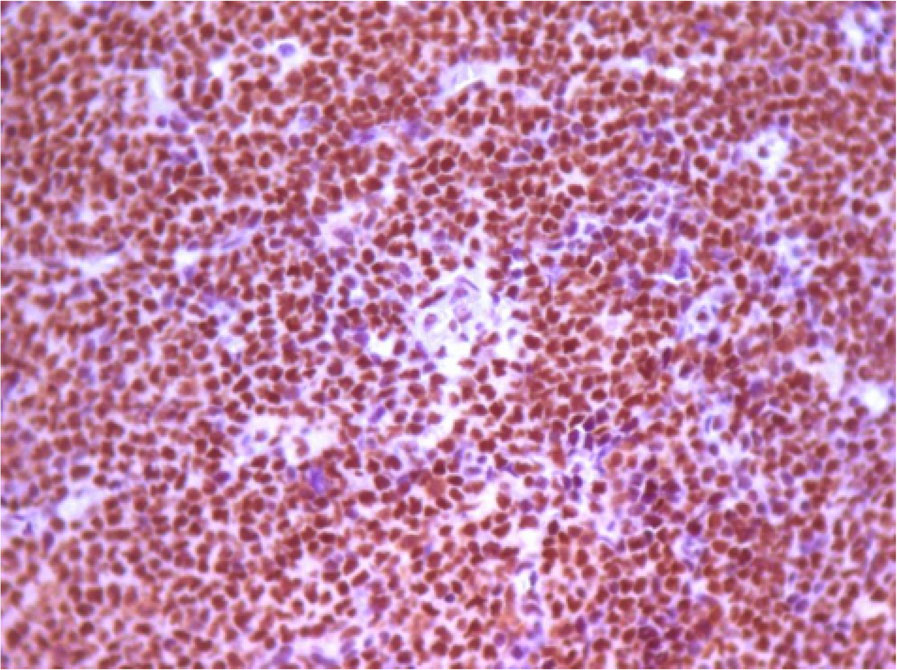
Immunohistochemical reaction showing strong positive expression (3+/3+) of the OCT-1 protein. Optical microscopy, 400x magnification

Bcl-2 protein was positive in 68.9% (40/58) of cases, and Oct-1 protein in 73.6% (42/57). In univariate analysis, Bcl-2 and Oct-1 were not associated with OS (*p* = 0.086) and PFS (*p* = 0.141). Bcl-2 protein was not associated with any clinical variable studied, however, there was a significant correlation between the expression of *BCL-2* gene expression ≥ median and Bcl-2 positivity by IHC (*p* < 0.001). Oct-1 protein did not show correlation with any clinical variable analyzed, but was significantly associated with age (*r* = 0.27; *p* = 0.01). The positivity of Oct-1 by IHC was not significantly associated with Oct-1 gene expression (*p* = 0.482). Figures 5, 6 and 7 demonstrate examples of immunohistochemical reactions for BCL-2 and OCT-1 proteins performed in this study.

### Multivariate analysis

In multivariate analysis, age ≥ 60 years (*p* = 0.006; [HR] 2.53; 95% CI: 1.31–4.89) and high aIPI (*p* < 0.001; [HR] 4.32; 95% CI: 2.01–9.32) were associated with worse OS. Age ≥ 60 years (*p* = 0.040; [HR] 2.13; 95% CI: 1.04–4.38), stage III and IV (*p* = 0.001; [HR] 4.47; CI 95% 1.82–10.96) and *OCT-1* gene expression ≥ median (*p* = 0.035; [HR] 2.22; 95% CI: 1.06–4.67) were associated with lower PFS.

## Discussion

In this study, we showed that overexpression of *OCT-1* gene was associated with shorter PFS in DLBCL treated with R-CHOP 21. In the subgroup analysis, *OCT-1* gene expression individualized a population with significant inferior prognostic within the high-risk aIPI cohort. Similar results were verified in the ≥60 years subgroup, where *OCT-1* expression equal to or higher than the median discriminated a population of worse prognosis. However, Oct-1 protein expression was not associated with PFS, or with OS in the same cohort. *BCL-2* gene expression and its protein expression were not correlated with prognosis in our study.

Taking into account our initial hypothesis, in which we supposed that the *OCT-1* effect would be dependent of *BCL-2* expression, the results obtained in this study were unexpected. On the other hand, *OCT-1* gene has been recognized as an oncogene, and it is overexpressed in various cancers [14], such as stomach [16], prostate [17], and cervical cancer [18]. *OCT-1* is broadly expressed in normal lymphocytes and in neoplastic cells of non-Hodgkin lymphoma and Hodgkin lymphoma [15, 20].

In agreement with other authors, our results confirmed that *BCL-2* expression is not associated with shorter survival in DLBCL treated with combination of Rituximab and chemotherapy [32–34]. Rituximab activates cell signals to modulate intracellular pathways that regulate proliferation and resistance, and potentiates the cytotoxicity of drugs and inhibits the expression of the antiapoptotic genes *BCL2/BCL-XL*. Rituximab also mediates the inhibition of p38 mitogen-activated protein kinase (MAPK), nuclear factor (NF)-kB, extracellular signal regulated kinase-1/2 (ERK-1/2), and Akt survival pathways [35]. Rituximab also significantly inhibits secretion and synthesis of IL-10, causing inhibition of cell-proliferation and *BCL-2* gene expression. In brief, IL-10 inhibition by Rituximab results in downregulation of the signal transducer and activator of transcription 3 (STAT3).

STAT3 inhibition reduces *BCL-2* gene expression, driving cells into apoptosis [35]. The active form of STAT3, pSTAT3, enhances tumor growth and tumor survival, cell migration, cell invasion, angiogenesis and downregulates the immune response. The STAT3 phosphorylation occurs directly or indirectly throughout activation of other intracellular signaling pathways, such as EGFR, HER2, Src, and JAK2 [35]. pSTAT3 is frequently found in many human cancers, including diffuse large B-cell lymphoma. Compared to germinal center B-cell-like lymphoma, the activated B-cell like subtype of DLBCL present poor response to current therapy and often exhibit overexpression or hyperactivation of STAT3 [36]. pSTAT3 overexpression is observed in 16% of DLBCL, and is associated with advanced disease, multiple extranodal sites of involvement, activated B-cell-like subtype, Myc protein expression and Myc/Bcl-2 protein expression [37]. Ok et al., also observed that pSTAT3 overexpression predicted inferior overall survival and progression-free survival in patients with de novo DLBCL [38].

Wang et al. demonstrated that hyperactivated STAT3 (pSTAT3) in esophageal cancer tissues correlated with overexpression of octamer transcription factor-1 gene. In this setting, high STAT3 phosphorylation determined shorter survival when compared with low STAT3 phosphorylation [37]. STAT3 inhibited apoptosis in esophageal squamous cells with high expression of *OCT-1* gene. In addition, high levels of pSTAT3 in normal human esophageal epithelium cells (HET-1A) elevated *OCT-1* gene expression and promoted proliferation and reduction of apoptosis. They also showed that STAT3 regulates the transcription and expression of *OCT-1* by directly targeting its promoter [37]. In this study, *STAT3* and *OCT-1* knock-out increased the expression of pro-apoptotic genes *BAX*, *BAD*, *CASPASE3*, and *CASPASE9,* and reduced the expression of antiapoptotic genes *BCL-2* and *BCL-XL* [37]. In an experiment using RNA interference technique and a lymphoma cell line harboring t(14;18), Heckman et al. demonstrated that downregulation of *OCT-1* and *OCT-2* gene expression reduced *BCL-2* gene expression and potentiated apoptosis [19].

All these data are in agreement with our results, that associated higher expression of *OCT-1* gene with poor prognosis in DLBCL. *OCT-1* gene also modulates expression of genes involved in cellular response to stress and DNA damage such as *H2B*, *TIF2*, *GnRH,* and related to B-cell immunoglobulin receptor. *OCT-1*-deficient fibroblasts are hypersensitive to radiation, doxorubicin and hydrogen peroxide and harbored elevated reactive oxygen species [39]. Expression of *OCT-1* gene is elevated in cells with DNA damage caused by inflammation and physical and chemical injuries [40, 41]. Zhao et al. [42] explored the cellular response to genotoxic stress in several human carcinoma cell lines, and showed significant improvement of *OCT-1* expression in cells exposed to antineoplastic agents. However, increment of mRNA after DNA injury was not detected, suggesting that induction of Oct-1 protein may be a posttranscriptional event [41, 42]. The induction of Oct-1 protein expression posttranscriptionaly does not require the normal cellular function of the tumor suppressor *P53*, indicating that the Oct-1 protein as a transcription factor, may play a role in *P53*-independent gene activation [42]. Different from *OCT-1* gene expression, we did not find any association between Oct-1 protein expression and prognosis. The lack of adequate samples to be processed and analyzed resulted in substantial missing data for protein expression. Nevertheless, in our cohort, the positivity of Oct-1 by IHC was not significantly associated with *OCT-1* gene expression (*p* = 0.482).

Interestingly, in our study, *OCT-1* gene overexpression individualized two distinct groups of prognosis inside the high-risk aIPI population. Patients with high aIPI and *OCT-1* overexpression can be classified as patients with higher risk (biological higher-risk). *OCT-1* gene expression might be used to identify candidates to receive more intensive treatments and conduct tests with new target drugs. Likewise, in patients older than 60 years, higher expression of *OCT-1* gene was associated with shorter PFS.

Our results support the importance of gene expression analysis as a strategy to identify new biomarkers in lymphoma, to improve the knowledge of lymphomagenesis and to find new targets for personalized therapy. The real-time PCR approach was used in this study due to its high sensitivity, capability of transcript quantification and to measure small changes in gene expression. In addition, it is affordable and easier to incorporate in the clinical practice. Compared with other methods such as microarray, gene expression by real time PCR, is less complex, cheaper and requires relatively small quantities of material obtained from paraffin tumor samples.

In this study, we standardized the real-time PCR following good practices, as recommended; all samples contained the same amount of RNA, two different genes of reference were used as internal control to normalize the data and the same calibrator (control) was used for all reactions [28]. Primers were designed to anneal to exon regions to prevent amplification of genomic DNA and its specificity was verified in the BLAST system. Furthermore, all patients undergone R-CHOP regimen and the histology was centrally reviewed to confirm a diagnosis of DLBCL.

## Conclusion

In conclusion, we demonstrated for the first time that the overexpression of *OCT-1* gene is an independent prognostic factor for progression-free survival in diffuse large B-cell lymphoma. Further studies are required to confirm our results.

## Data Availability

All data generated and analysed during this study are included in this published article. The raw data for this study are in the possession of the correspondence author and may be made fully available in the event of a request to the correspondence author via e-mail.

## Abbreviations

DLBCL: Diffuse large B-cell lymphoma
RNA: Ribonucleic acid
RT-PCR: Real time-polymerase chain reaction
ORR: Overall response rate
OS: Overall survival
PFS: Progression-free survival
NHLs: Non-Hodgkin lymphomas
R-CHOP: Rituximab, cyclophosphamide, doxorubicin, vincristine and prednisone
DA-REPOCH: Dose adjusted- rituximab, etoposide, prednisone, vincristine, cyclophosphamide and doxorubicin
PMBCL: Primary mediastinal B-cell lymphoma
IPI: International Prognostic Index
NCCN-IPI: National Compreensive Cancer Network-International Prognostic Index
OCT-1: Octamer transcription factor 1
DNA: Desoxiribonucleic acid
BCL-2: B-cell lymphoma 2 gene
WHO: World Health Organization
CT: Computerized tomography
IWRC: International Workshop to Standardize Response Criteria for Non-Hodgkin’s Lymphoma
LDH: Lactate dehydrogenase
H&E: Hematoxylin-Eosin
GC: Germinal center
ABC: Activated B-cells
IHC: Immunohistochemistry
FFPE: Formalin-fixed paraffin-embedded
NCBI: National Center for Biotechnology Information
HR: Hazard ratio
CR: Complete response
PR: Partial response
ECOG: Eastern Cooperative Oncology Group
MAPK: Mitogen-activated protein kinase
NF-kB: Nuclear factor kappa B
ERK-1/2: Extracellular signal regulated kinase 1/2
STAT3: Signal transducer and activator of transcription 3
HET-1A: Human esophageal epithelium cells 1A
